# Case Report: Giant acute hemorrhagic colloid cyst in the septum pellucidum-foramen of Monro region with obstructive hydrocephalus in an 8-month-old infant

**DOI:** 10.3389/fonc.2026.1899449

**Published:** 2026-09-16

**Authors:** Peng Peng, Yuecheng Zeng, Zhongyin Guo, Chunhua Wang

**Affiliations:** 1 Department of Neurosurgery, Xiangyang Central Hospital, Affiliated Hospital of Hubei University of Arts and Science, Xiangyang, China; 2 Department of Central Laboratory, Xiangyang Central Hospital, Affiliated Hospital of Hubei University of Arts and Science, Xiangyang, Hubei, China; 3 Department of Clinical Laboratory, Xiangyang Central Hospital, Affiliated Hospital of Hubei University of Arts and Science, Hubei, Xiangyang, China

**Keywords:** foramen of Monro, hemorrhagic colloid cyst, infant, obstructive hydrocephalus, pediatric neurosurgery, septum pellucidum, third ventricle

## Abstract

Colloid cysts are uncommon benign neuroepithelial cystic lesions that typically arise in the anterior third ventricle near the foramen of Monro. Pediatric colloid cysts are rare, and giant hemorrhagic lesions centered in the septum pellucidum-foramen of Monro region have only rarely been reported in infancy. We report the case of an 8-month-old boy who presented with a 1-day history of sudden bulging of the anterior fontanelle, recurrent vomiting, and decreased responsiveness. Cranial magnetic resonance imaging revealed a giant cystic mass extending from the septum pellucidum–foramen of Monro region to the anterior third ventricle, measuring approximately 38 mm × 23 mm × 35 mm. The lesion showed mildly hyperintense signal on T1-weighted and FLAIR sequences, with a focal fluid–fluid level and a T2-hypointense clot-like nodule. Supratentorial ventricular dilatation and periventricular interstitial edema were also observed, indicating acute obstructive hydrocephalus. The patient underwent emergency microsurgical decompression, evacuation of intracystic fluid and hematoma, sampling of wall-like tissue, septostomy, and external ventricular drainage. Intraoperatively, dark-brown intracystic fluid mixed with abundant blood clots was identified. Histopathological examination showed wall-like fibrovascular tissue with edema, hemorrhage, necrosis, and focal calcification. After pediatric intensive care support, anti-infective therapy, and symptomatic management, the patient recovered well. Follow-up MRI at 3 months after surgery showed no definite evidence of residual or recurrent lesion. This case suggests that infant who cannot verbalize headache, bulging of the anterior fontanelle, postprandial vomiting, and decreased responsiveness should be regarded as important warning signs of acute intracranial hypertension. Early radiological recognition, timely relief of cerebrospinal fluid pathway obstruction, and standardized perioperative intensive care are critical for improving clinical outcomes.

## 1 Introduction

Colloid cysts are generally regarded as benign cystic lesions arising from neuroepithelial or endodermal-like epithelium. They account for approximately 0.5%–1.0% of intracranial tumors or space-occupying lesions and are most commonly located in the anterior third ventricle, near the foramen of Monro. Because this region lies adjacent to the cerebrospinal fluid pathway between the bilateral lateral ventricles and the third ventricle, even a relatively small lesion may produce intermittent or acute obstructive hydrocephalus through a “ball-valve” mechanism at the foramen of Monro (1–3).

Pediatric colloid cysts are substantially less common than their adult counterparts, and previously reported pediatric cases have predominantly involved school-aged children or adolescents. Multicenter data indicate that most symptomatic pediatric patients present after 10 years old, whereas cases occurring in infancy have been reported only sporadically (4–9). Acute intracystic hemorrhage has also been reported rarely; it may rapidly increase cyst volume, abruptly compress the foramen of Monro, and cause acute hydrocephalus, impaired consciousness, or sudden death (10–18). Beyond the classic third ventricular location, colloid cysts involving the septum pellucidum, cavum septi pellucidi, or cavum Vergae have been described in only a limited number of reports and may mimic cavum septi pellucidi cysts, ependymal cysts, or other midline intraventricular lesions (11–14, 19–22).

Here, we report a case of clinically and radiologically supported a giant acute hemorrhagic colloid cyst in the septum pellucidum–foramen of Monro region with obstructive hydrocephalus in an 8-month-old infant. In conjunction with the published literature, we discuss this rare case from the perspectives of early age at onset, unusual anatomical location, hemorrhage-related acute deterioration, the radiological–pathological diagnostic evidence chain, differential diagnosis, and surgical strategy, with the aim of informing the early recognition and management of similar rare presentations.

## 2 Case report

An 8-month-old boy was transferred to the Department of Neurosurgery of a tertiary hospital in Xiangyang, Hubei Province, China, on September 16, 2025, after cranial computed tomography at an outside hospital suggested a space-occupying lesion in the corpus callosum/septum pellucidum region with hemorrhage and obstructive hydrocephalus (**Figure 1A**). One day before admission, the child developed bulging of the anterior fontanelle, repeated vomiting after feeding, and decreased responsiveness. He had previously been healthy. His medical history was otherwise unremarkable, with no documented history of surgery, trauma, infectious disease, chronic underlying illness, or known hereditary disorder. A separate psychosocial history was not applicable because of his age. He had a known history of penicillin allergy, and preoperative cephalosporin skin testing was positive.

**Figure 1:**
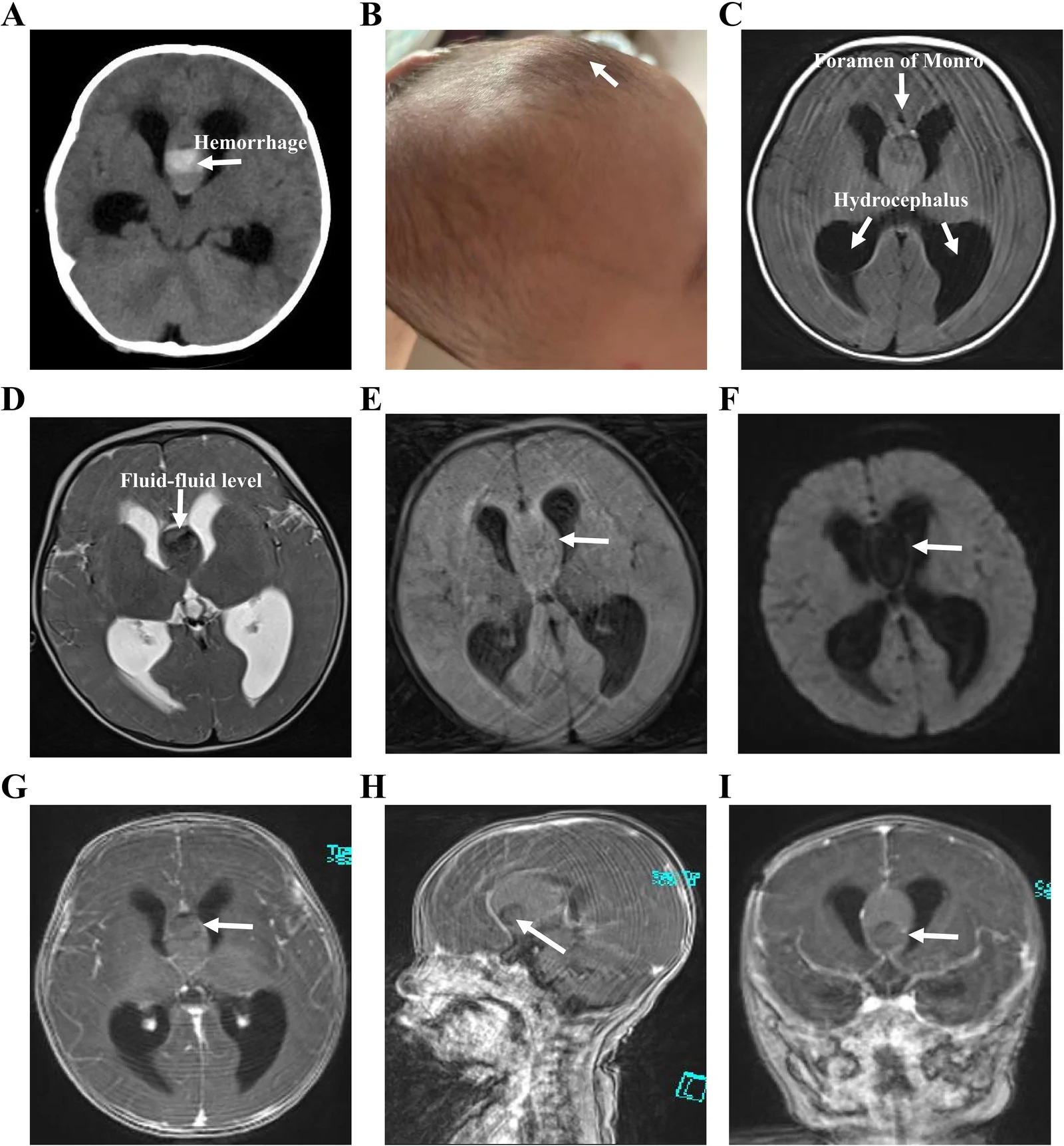
Preoperative clinical presentation and imaging findings. **(A)** Preoperative CT revealed a hyperdense hemorrhagic component in the septum pellucidum–foramen of Monro region (labeled “Hemorrhage”), with acute obstructive hydrocephalus. **(B)** Marked bulging of the anterior fontanelle. **(C)** Axial T1-weighted MRI showed the lesion centered adjacent to the foramen of Monro (labeled “Foramen of Monro”) and enlarged lateral ventricles (labeled “Hydrocephalus”). **(D)** Axial T2-weighted MRI demonstrated a fluid–fluid level and T2-hypointense clot-like nodule (labeled “Fluid-fluid level”). **(E)** Axial FLAIR showed heterogeneous signal and periventricular interstitial edema. **(F)** DWI showed no definite diffusion restriction. **(G-I)** Post-contrast axial **(G)**, sagittal **(H)** and coronal **(I)** MRI showed mild focal rim enhancement around the anteroinferior hypointense component. White arrows and labels identify the principal findings.

On admission, his body temperature was 36.5 °C, pulse rate 80–89 beats/min, respiratory rate 15 breaths/min, blood pressure 101/66 mmHg, and body weight 9 kg. He appeared weak and mildly irritable, with marked outward bulging of the anterior fontanelle (**Figure 1B**). Both pupils were equal and round, approximately 3 mm in diameter, and briskly reactive to light. Spontaneous movement of all four limbs was preserved, muscle tone was not increased, bilateral pathological reflexes were negative, and no obvious meningeal irritation signs were observed. Laboratory testing showed no evidence of coagulation dysfunction. Complete blood count revealed a white blood cell count of 11.32 × 10^9^/L, hemoglobin level of 116 g/L, and platelet count of 457 × 10^9^/L. Biochemical tests showed only nonspecific abnormalities, including mild hyponatremia (Na^+^, 134.6 mmol/L).

Cranial magnetic resonance imaging, including plain, contrast-enhanced, and diffusion-weighted sequences, demonstrated a cystic lesion extending from the septum pellucidum/foramen of Monro region to the anterior third ventricle, measuring approximately 38 mm × 23 mm × 35 mm, with well-defined margins (**Figure 1C**). The lesion showed mildly hyperintense signal on T1-weighted and FLAIR sequences, with heterogeneous signal in the anteroinferior portion, anterior layering, and a nodular T2-hypointense component; a fluid–fluid level and imaging signs of hemorrhage were identified (**Figures 1C–G**). The mild T1 hyperintensity was interpreted as reflecting concentrated proteinaceous material and/or subacute blood products. The fluid–fluid level suggested dependent layering of blood products, whereas the T2-hypointense nodule was considered a retracted clot or hemosiderin-rich component. No obvious diffusion restriction was seen on DWI (**Figure 1F**), and mild focal rim enhancement was observed around the anteroinferior hypointense nodule after contrast administration (**Figures 1G-I**). The rim enhancement was interpreted cautiously as possible vascularized cyst wall or reactive inflammation rather than as evidence of a substantial solid tumor component (16, 23–25). The lesion was associated with supratentorial ventricular dilatation and mild periventricular interstitial edema adjacent to both lateral ventricles, while the midline structures remained centered. Based on the foramen of Monro–centered anatomy, non-CSF signal characteristics, hemorrhagic layering, lack of diffusion restriction, and acute hydrocephalus, a hemorrhagic colloid cyst was considered the leading diagnosis.

Given the presence of a giant midline intraventricular mass, acute obstructive hydrocephalus, and progressive signs of intracranial hypertension, the indication for emergency surgery was clear. On September 17, 2025, under general anesthesia, the patient underwent microsurgical decompression and resection of the deep cystic lesion, intracystic hematoma evacuation, septostomy, and external ventricular drainage. Intraoperatively, the lesion was cystic, with its inferior aspect protruding into the third ventricle (**Figure 2A**). The cyst contained dark-brown fluid mixed with abundant blood clots (**Figure 2B**). After evacuation of the cyst fluid and clots, solid intracystic/cyst wall-like tissue was resected totally and submitted for pathological examination. Grossly, the specimen consisted of a single gray-brown tissue fragment measuring 0.6 cm × 0.5 cm × 0.2 cm (**Figure 2C**). Microscopically, fibrovascular tissue with edema, hemorrhage, necrosis, focal calcification, scattered chronic inflammatory cell infiltration, and a focal wall-like structure was observed (**Figure 2D**). Integrating the intraoperative findings, and imaging features, the diagnosis was mainly considered as a clinically and radiologically supported giant colloid cyst of the septum pellucidum–foramen of Monro region with acute hemorrhage and obstructive hydrocephalus.

**Figure 2:**
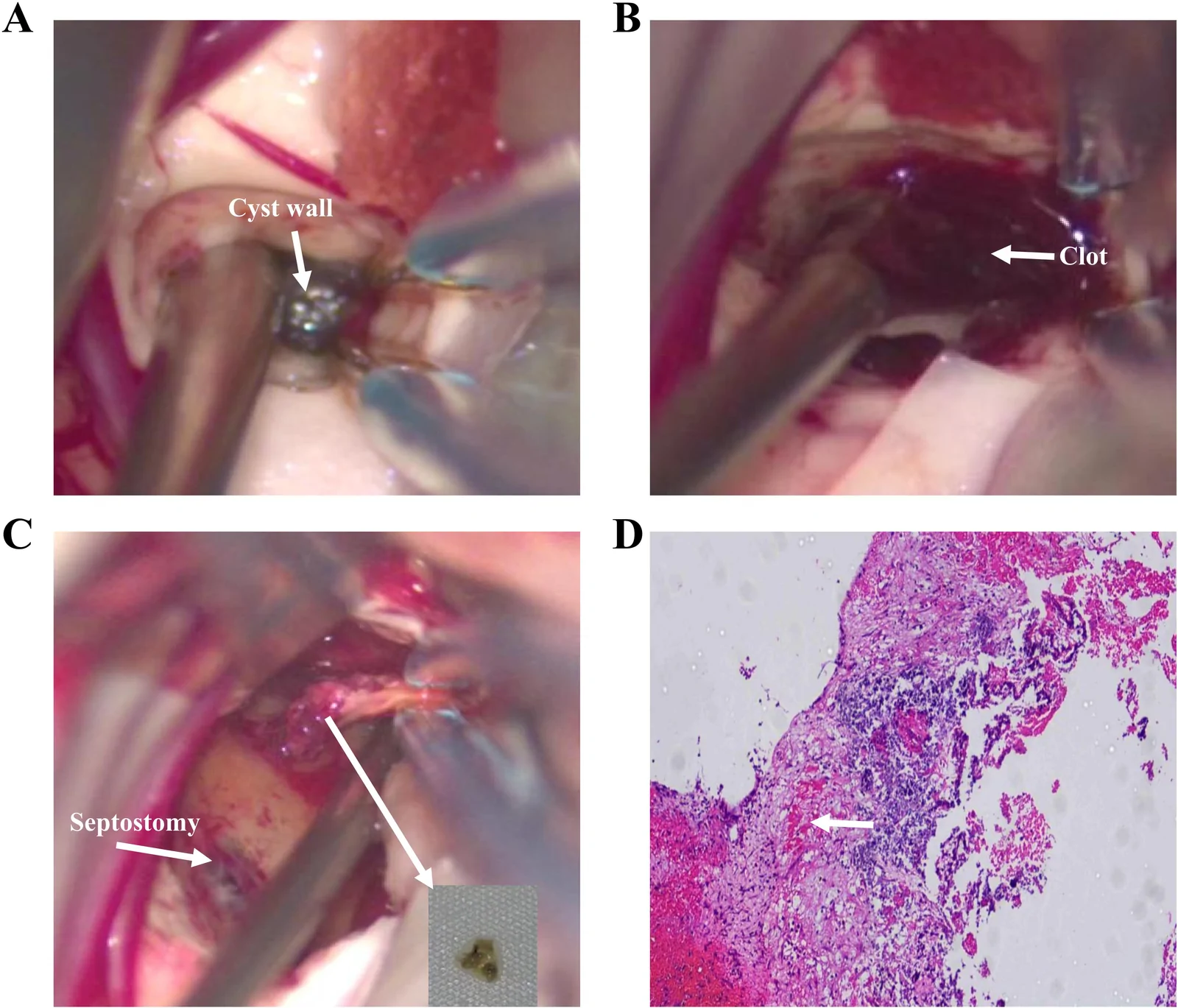
Intraoperative cyst characteristics and histopathological findings. **(A)** The cyst wall was visualized after incision of the corpus callosum (labeled “Cyst wall”). **(B)** Dark-brown intracystic fluid and abundant clot were encountered (labeled “Clot”). **(C)** The septostomy is indicated (label), and the inset shows the resected gray-brown wall-like tissue. **(D)** Hematoxylin and eosin staining of the cyst wall showed fibrovascular tissue with hemorrhage. White arrows and labels identify the indicated structures.

Postoperatively, the child was transferred to the pediatric intensive care unit. In the early postoperative period, he developed high fever, with a maximum temperature of 39.7 °C, thick sputum, coffee-ground gastric drainage, and transient brown urine. Comprehensive treatment was administered, including respiratory support, sedation and analgesia, gastric protection, fluid replacement, urine alkalinization, and anti-infective therapy. Because of penicillin and cephalosporin allergy, the perioperative anti-infective regimen was adjusted sequentially according to the clinical condition and drug-allergy profile. During postoperative recovery, the subcutaneous drainage tube and external ventricular drain were removed in sequence. After removal of the external ventricular drain, transient recurrent vomiting occurred; repeat cranial CT showed no obvious worsening of intracranial hypertension (**Figure 3A**), and the symptoms resolved after reducing milk intake and providing fluid supplementation. On October 2, 2025, the patient was discharged with normal body temperature, satisfactory feeding, no vomiting, clear consciousness, and near-normalization of infection-related indicators. At 3 months after surgery (December 22, 2025), no bulging of the anterior fontanelle was observed (**Figure 3B**), and MRI showed postoperative changes, absorption of the previous bilateral frontotemporal subdural effusions, and no definite evidence of residual or recurrent lesion (**Figures 3C, D**).

**Figure 3:**
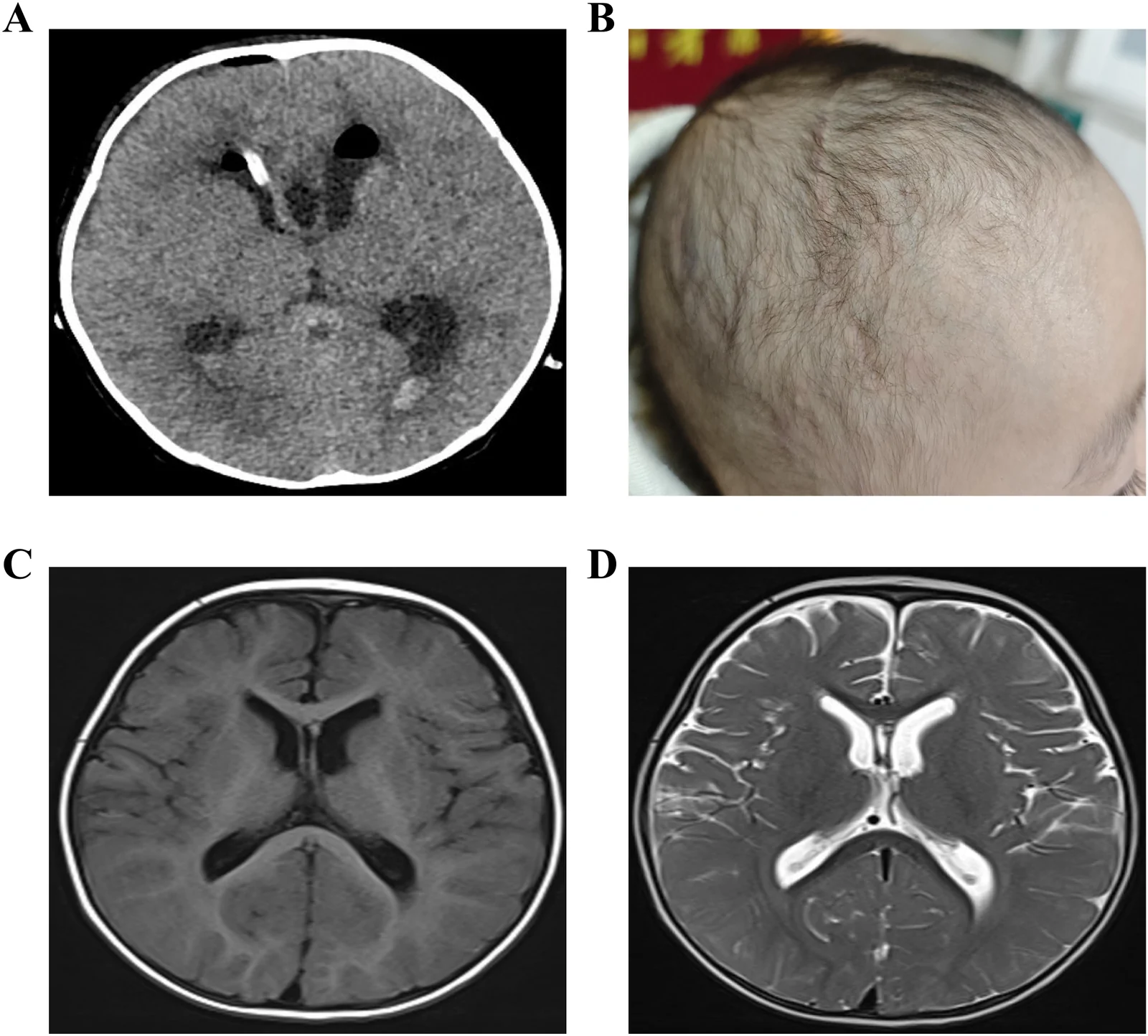
Postoperative clinical presentation and imaging findings. **(A)** Postoperative CT after removal of the external ventricular drain showed postoperative decompression without recurrent worsening of hydrocephalus. **(B)** At 3 months, the anterior fontanelle was no longer bulging. **(C, D)** Follow-up MRI at 3 months showed postoperative changes, resolution of the previous subdural effusions, and no definite residual or recurrent lesion.

## 3 Discussion

Colloid cysts are benign developmental epithelial cysts that most frequently arise in the anterior third ventricle near the foramen of Monro; overall, they are rare (**Table 1**). Adult colloid cysts are usually detected between 30–60 years old, and clinical manifestations range from incidental discovery to headache, vomiting, memory impairment, impaired consciousness, or sudden death (1–3, 17, 18). Although colloid cysts may occur in children, they remain uncommon, and most pediatric series have involved patients older than 6 years or beyond school age (4–8). Burgess and Jayamohan reported a nonhemorrhagic third ventricular colloid cyst in a 12-week-old infant that was managed with endoscopic third ventriculostomy and cyst fenestration (9), whereas Farooq et al. described acute hemorrhage in a 9-year-old child (10). Septum pellucidum and cavum-related giant lesions have primarily been reported in adults or small series (11–16). Compared with these reports, the present case combined infancy, a septum pellucidum–foramen of Monro epicenter, a 38-mm lesion, acute hemorrhage, and obstructive hydrocephalus. This combination is rare, but the present report does not establish a unique disease phenotype.

**Table 1:** Representative cases and studies of colloid cysts involving the septum pellucidum–foramen of monro/third ventricular region.

Reference	Case/Study Population	Focus Content	Findings
Alnaghmoosh et al. (4); Maqsood et al. (5)	Pediatric third ventricular colloid cyst case series	Pediatric epidemiology	Pediatric colloid cysts are uncommon overall, and most reported cases do not occur during infancy.
Kumar et al. (6); Vazhayil et al. (7); Roth et al. (8)	Pediatric colloid cyst surgical series/multicenter study	Treatment strategy and outcomes	Symptoms in pediatric patients are frequently related to hydrocephalus, and the surgical approach should be individualized.
Burgess et al. (9)	Third ventricular colloid cyst in a 12-week-old infant	Infantile case	Colloid cysts can occur in infancy; however, this case was not complicated by acute hemorrhage.
Farooq et al. (10)	Hemorrhagic colloid cyst in a 9-year-old child	Pediatric hemorrhagic lesion	Hemorrhage may cause acute hydrocephalus and require emergency management
Ciric and Zivin (11); Sadashiva et al. (12)	Colloid cysts of the septum pellucidum	Ectopic/unusual anatomical location	Lesions in the septum pellucidum are rarely reported, and intraoperative localization and complete excision may be more challenging.
Pryce et al. (13); Ashirov et al. (14)	Giant colloid cysts in the cavum septi pellucidi/cavum Vergae in adults	Giant lesions involving septum pellucidum-related cavities	Septum pellucidum-related cavities may accommodate large cystic lesions before overt cerebrospinal fluid pathway obstruction occurs.
Westwick et al. (15); Al Abdulsalam et al. (16)	Hemorrhagic/ruptured colloid cysts	Acute hemorrhage	Hemorrhage may alter imaging characteristics and precipitate acute hydrocephalus.
Sheikh et al. (28); Boogaarts et al. (29)	Systematic review of surgical approaches/long-term outcomes after endoscopic treatment	Comparison of endoscopic and microsurgical approaches	Endoscopy is minimally invasive, but the extent of resection, recurrence risk, and complications should be weighed comprehensively.
Siala et al. (19); Ciołkowski et al. (20)	Reviews of septum pellucidum imaging and variant cavities	Differential diagnosis	The cavum septi pellucidi and cavum Vergae are common developmental variants; cystic enlargement should be differentiated from pathological cysts.
Present case	Giant hemorrhagic colloid cyst in an 8-month-old boy	Infantile case	A rare case combination: infancy, a septum pellucidum–foramen of Monro epicenter, a 38-mm lesion, acute hemorrhage, and obstructive hydrocephalus, which was successfully treated by microsurgical resection, with a favorable outcome.

The septum pellucidum and its variant cavities represent an easily overlooked anatomical compartment within the anterior midline ventricular system. Colloid cysts of the septum pellucidum were first reported by Ciric and Zivin, and only a limited number of adult cases or small case series have subsequently been published (11, 12). The giant colloid cyst occupying the cavum septi pellucidi et vergae reported by Pryce et al. and the giant cavum septi pellucidi colloid cyst reported by Ashirov et al. suggest that septum pellucidum-related cavities may provide a concealed space for cystic expansion, allowing lesions to reach a considerable size before overt cerebrospinal fluid obstruction develops (13, 14). In the present case, the lesion was centered in the septum pellucidum–foramen of Monro region, protruded into the anterior third ventricle, and measured approximately 38 mm at its maximum diameter. Its large size and hemorrhagic expansion may have jointly precipitated acute obstruction.

Hemorrhage provides a plausible pathophysiological explanation for the acute deterioration in this case. Colloid cysts usually enlarge slowly; however, intracystic hemorrhage may rapidly increase cyst volume and generate clot-like contents, producing sudden compression or obstruction of the foramen of Monro (10, 15, 16). Reported hemorrhagic colloid cysts have mostly occurred in adults or older children, with presentations including sudden headache, vomiting, impaired consciousness, acute obstructive hydrocephalus, and, in severe cases, sudden death (10, 15–18). The present infant could not verbalize headache, but the rapid onset of a bulging anterior fontanelle, post-feeding vomiting, and decreased responsiveness within 1 day represented clinically important signs of raised intracranial pressure in infancy. The intraoperative dark-brown fluid and abundant clots, together with the MRI fluid–fluid level and T2-hypointense nodule, supported acute/subacute intracystic hemorrhage, although none of these findings is individually pathognomonic.

The imaging appearance of colloid cysts varies with the relative proportions of protein, cholesterol, water, and hemorrhagic products. T1 hyperintensity may reflect concentrated proteinaceous material, cholesterol-rich contents, or subacute blood products; a fluid–fluid level indicates layering of components with different physical properties, commonly blood products; and a T2-hypointense nodule may represent a retracted clot or hemosiderin-rich component. Mild rim enhancement can reflect vascularized capsule or reactive inflammation, but it is nonspecific and does not by itself establish a neoplastic solid component (16, 23–25). Histologically, colloid cysts are typically lined by simple to pseudostratified cuboidal or columnar epithelium, often with ciliated and goblet cells (23, 24). In this case, the small specimen showed fibrovascular wall-like tissue with hemorrhage, necrosis, and calcification, but no diagnostic epithelial lining or colloid material. This is the principal limitation of the report. Hemorrhagic destruction and limited sampling may account for nonvisualization of the epithelial lining. However, sampling error cannot be assumed, and alternative hemorrhagic intraventricular cysts cannot be completely excluded. Therefore, the diagnosis should not rely solely on histological sections, but should instead be established through an integrated evidence chain consisting of lesion anatomy, MRI signal characteristics, intraoperative dark-brown cyst fluid and blood clots, and a wall-like pathological structure.

In infants and young children, cystic lesions of the septum pellucidum, foramen of Monro, or anterior third ventricle should be differentiated from cavum septi pellucidi cysts, ependymal cysts, choroid plexus cysts, midline germ cell tumors, central neurocytomas, teratomas, and other hemorrhagic intraventricular lesions (19, 20, 26, 27). A cavum septi pellucidi cyst typically follows cerebrospinal-fluid signal on all sequences and produces outward bowing of the septal leaflets; an ependymal cyst is usually thin-walled, nonenhancing, and CSF-like; and choroid plexus cysts are generally attached to the choroid plexus and lack a foramen of Monro–centered proteinaceous appearance. Hemorrhagic tumors more often contain a substantial enhancing solid component, diffusion restriction, infiltrative change, or tumor-associated perfusion characteristics. In the present case, the features that most strongly favored a clinically and radiologically supported hemorrhagic colloid cyst were the foramen of Monro–centered epicenter, T1/FLAIR signal higher than CSF, a fluid–fluid level, a T2-hypointense clot-like nodule, absence of diffusion restriction, lack of a substantial enhancing solid component, and the operative finding of dark-brown viscous cyst fluid with abundant clots.

The surgical approach for pediatric colloid cysts should be individualized according to patient age, ventricular size, lesion location, viscosity of cyst contents, hemorrhage, and clot burden. Systematic reviews and large surgical series indicate that endoscopy is less invasive and may shorten hospitalization, whereas microsurgery is associated with higher rates of complete resection and lower rates of recurrence or reoperation (28–33). Endoscopy is generally most suitable when the lesion is classically located, small to medium in size, and accessible through a safe ventricular corridor. In the present case, endoscopy was not selected because the lesion measured 38 mm, contained abundant organized hematoma, required direct clot evacuation and hemostasis, had an uncertain tissue diagnosis requiring adequate sampling, and occurred in an infant in whom a limited working corridor and planned septostomy increased technical complexity. Microsurgery combined with external ventricular drainage and septostomy therefore better addressed the immediate goals of decompression, clot removal, tissue sampling, hemostasis, and restoration of cerebrospinal fluid circulation (24, 28). Given the long life expectancy of pediatric patients and the potential for long-term recurrence after residual cyst wall or incomplete resection, MRI follow-up is recommended at 3–6 months, at 1 year, and periodically thereafter, together with continued assessment of head circumference, growth and development, neurocognitive status, and symptoms related to hydrocephalus.

This case illustrates a rare combination of infancy, a giant hemorrhagic cystic lesion centered in the septum pellucidum–foramen of Monro region, and acute obstructive hydrocephalus. In infants who cannot verbalize headache, bulging of the anterior fontanelle, recurrent vomiting, and decreased responsiveness should raise strong suspicion for acute intracranial hypertension. Hemorrhage, necrosis, and sampling limitations may obscure the typical radiological and pathological features of colloid cysts; therefore, diagnosis should rely on an integrated assessment of clinical presentation, anatomical location, MRI findings, intraoperative observations, and histopathology. For cases complicated by acute hydrocephalus, substantial clot burden, or unusual location, timely microsurgical decompression/resection, clot evacuation, restoration of cerebrospinal fluid pathways, and perioperative intensive care may achieve favorable short-term outcomes; nevertheless, long-term radiological and neurodevelopmental follow-up remains essential.

## Data Availability

The original contributions presented in the study are included in the article/supplementary material. Further inquiries can be directed to the corresponding author.
